# Normal vibration distribution search-based differential evolution algorithm for multimodal biomedical image registration

**DOI:** 10.1007/s00521-023-08649-z

**Published:** 2023-05-30

**Authors:** Peng Gui, Fazhi He, Bingo Wing-Kuen Ling, Dengyi Zhang, Zongyuan Ge

**Affiliations:** 1 School of Computer Science, Wuhan University, Wuhan, 430072 People’s Republic of China; 2 School of Information Engineering, Guangdong University of Technology, Guangzhou, 510006 People’s Republic of China; 3 AIM Lab, Faculty of IT, Monash University, Melbourne, VIC 3800 Australia; 4 Monash-Airdoc Research, Monash University, Melbourne, VIC 3800 Australia

**Keywords:** Medical image registration, Optimization, Metaheuristic, Bernstein search differential evolution algorithm

## Abstract

In linear registration, a floating image is spatially aligned with a reference image after performing a series of linear metric transformations. Additionally, linear registration is mainly considered a preprocessing version of nonrigid registration. To better accomplish the task of finding the optimal transformation in pairwise intensity-based medical image registration, in this work, we present an optimization algorithm called the normal vibration distribution search-based differential evolution algorithm (NVSA), which is modified from the Bernstein search-based differential evolution (BSD) algorithm. We redesign the search pattern of the BSD algorithm and import several control parameters as part of the fine-tuning process to reduce the difficulty of the algorithm. In this study, 23 classic optimization functions and 16 real-world patients (resulting in 41 multimodal registration scenarios) are used in experiments performed to statistically investigate the problem solving ability of the NVSA. Nine metaheuristic algorithms are used in the conducted experiments. When compared to the commonly utilized registration methods, such as ANTS, Elastix, and FSL, our method achieves better registration performance on the RIRE dataset. Moreover, we prove that our method can perform well with or without its initial spatial transformation in terms of different evaluation indicators, demonstrating its versatility and robustness for various clinical needs and applications. This study establishes the idea that metaheuristic-based methods can better accomplish linear registration tasks than the frequently used approaches; the proposed method demonstrates promise that it can solve real-world clinical and service problems encountered during nonrigid registration as a preprocessing approach.The source code of the NVSA is publicly available at https://github.com/PengGui-N/NVSA.

## Introduction

Image registration [1, 2] is the basis of medical image analysis; this task can establish consistency among the corresponding anatomical structures in different medical images in space and is also a key technology for realizing precision medicine [3]. These attributes cause medical image registration to play an important role in clinical applications such as precise disease diagnosis [4], atlas analysis [5], image-guided radiotherapy [6] and surgical navigation [7]. Until recently, image registration was mostly performed manually by clinicians. However, many registration tasks can be quite challenging, and the quality of manual alignments is highly dependent upon the expertise of the user, which can be clinically disadvantageous [8]. These issues make automated registration methods urgently needed for clinical use.

During the preprocessing work of multimodal image fusion [9], multimodal image registration aligns multimodal information into the same image, making it easier for doctors to more accurately observe lesions and structures as detailed diagnostic information. The registrations of dynamic images collected at different times and the changes in lesions and organs can be quantitatively analyzed, making medical diagnosis, surgical planning, and radiation therapy planning more accurate and reliable. Since different modalities exhibit different characteristics, finding fast and accurate correspondences between images with different modalities is still a challenge.

In multimodal registration, the greatest difficulty comes from the great variability exhibited by organ and tissue appearances when imaged with different physical principles, which results in the lack of a general rule for establishing structure correspondence [10]. In this case, finding a proper similarity measure can be regarded as the central part of the objective function, making it the most important and challenging step. Particularly, the similarity measure between the images to be registered consists of numerous local extrema, making multimodal image registration an optimization problem. These aspects consequentially make optimization algorithms crucial parts of image registration.

Many optimization algorithms have been applied to multimodal registration. Compared to the traditional local search methods such as Powell’s method or the Levenberg-Marquardt (LM) method, metaheuristics-based methods [11–15] are excellent in that they rarely fall into local extrema, stably obtain global optima, are robust, and exhibit insensitivity to noise disturbances. Many metaheuristic algorithms have been applied in the past decade as optimization methods for medical image registration [16]. Regardless, metaheuristic algorithms are usually employed in situations where suboptimal solutions can be easily found but optimal solutions cannot be obtained in a stable manner [17–19]. One of the reasons for this phenomenon is that metaheuristic algorithms are usually limited to the initial values of the transformation parameters, which often lead the entire optimization process to fall into local extrema. Another reason is that in the latter stage of the registration process, the optimization procedure requires the algorithm to have good local search capabilities to obtain the optimal transformation matrix. These inner characteristics of intensity-based methods result in high requirements for the utilized optimization algorithm. The algorithm must have good global and local search capabilities, and it requires a good balance between exploration and exploitation [20].

As some of the most common metaheuristic algorithms, differential evolution (DE) and its variants have been applied to solve various optimization problems [21]. However, the directly application of DE to medical image registration still encounters many problems. For example, the medical image registration task is very demanding in terms of exploitation, which is often used to measure the pros and cons of the employed image registration optimization algorithm. Based on that, the most important shortcomings faced when applying DE to medical image registration are as follows. (1) DE occasionally falls into local minima, making DE unable to stably converge to the optimal region. (2) DE is capable of finding suboptima but incapable of attaining optimum values. In this case, it is necessary to replace DE with other modified algorithms to better complete the registration task.

Civicioglu et al. [22] proposed a modified DE method, named the Bernstein search-based DE (BSD) algorithm, by adopting 2nd-degree Bernstein polynomials that cooperate with a random crossover process. It has been proven that BSD is outperformed by approaches such as the artificial bee colony (ABC) algorithm [23], adaptive differential evolution (JADE) algorithm [24], cuckoo search algorithms (CUCKOO) [25], and weighted differential evolution (WDE) algorithm [26] in numerical function optimization and image evolution problems. However, BSD takes 2nd Bernstein polynomials as the crossover ratio, so this search strategy possesses some shortcomings. (1) The 2nd Bernstein polynomial curve is constant, which is not conducive to the variability of the algorithm. (2) The search pattern of BSD is not suitable for medical image registration, especially in the later exploitation stage of the registration process. Hence, we propose a modified BSD algorithm, a highly efficient metaheuristic named the normal vibration distribution search-based differential evolution algorithm (NVSA), to fix this problem.

The NVSA utilizes a new crossover method and search strategy. These improvements help the proposed method improve both its exploitation and exploration abilities and further form a balance between them in medical image registration scenarios. The original intention of the design of this method is to solve the problems that metaheuristic algorithms consume too many computation resources and achieve low precision. In addition, two-dimensional linear registration needs to optimize three to six parameters, which enables the NVSA to complete the registration of rigid and similar transformations without preregistration.

In the last decade, researchers have tended to use some classic approaches such as ANTS [27], Elastix [28] and FSL [29] to complete simple registration tasks. The advantage of these classic applications is that they do not require pretraining to quickly obtain results. Many deep learning methods also tend to use the above software to perform the registration task as a preprocessing step (linear) for deep learning (nonlinear) [30–32]. Convenient as these classic tools are, their accuracy still leaves much to be desired. Conversely, the proposed method can better complete the registration task with acceptable time consumption. Therefore, the NVSA has the potential to replace the commonly used linear registration method as a state-of-the-art (SOTA) approach.

We experimentally compare the proposed method, referred to as the NVSA, with nine benchmark metaheuristic algorithms and three frequently used applications on computed tomography (CT) and magnetic resonance imaging (MRI) brain images. The results show that the NVSA can achieve optimal accuracy at a reasonable computational cost, and more importantly, it outperforms the benchmark algorithms and classic registration methods. We further utilize Friedman’s mean rank test [33] and Bonferroni correction-adjusted significance to statistically prove that the results are not obtained by chance. The experimental results further suggest that different problem instances require different proportions of exploitation and exploration. Both conducted experiments confirm the importance of our new adaptive approach underpinned by the proposed mutation methods in the NVSA algorithm. The main contributions of our work are summarized as follows.

- We propose a new metaheuristic algorithm called the NVSA, which is modified from BSD by replacing the $2^{n}$ Bernstein polynomials with a novel normal vibration distribution and coordinating the approach with a variable search pattern.
- We test the NVSA with five other metaheuristic algorithms on 23 classic optimization functions and summarize them with their accuracy, robustness and significance level.
- We test the NVSA with five other metaheuristic algorithms on 16 brain images derived from real-world patients with 41 different type of multimodal registration and summarize them with their accuracy, time consumption, robustness and statistics. Moreover, we present supplementary figures including histograms for the test images, visualizations of the registration process, and population quality figures based on the number of iterations.
- We test the proposed algorithm with the three other classic medical image registration methods that are frequently used in various medical image registration scenarios. The experimental results prove that the proposed method has the potential to surpass and replace the existing classic registration tools in linear registration tasks.
- The present study demonstrates that the application of metaheuristic-based methods can effectively improve the accuracy and efficacy of linear registration tasks over that achieved with classic methods. These findings indicate that our work holds great promise for addressing the practical clinical needs of nonrigid registration as a preprocessing procedure.

The remainder of this paper is organized as follows. In Sect. 2, related works are presented. The basic BSD algorithm and the innovations and working principles of the NVSA are described in Sect. 3. Then, Sect. 4 demonstrates experiments involving numerical function-based optimization problems. Section 5 indicates the experimental results obtained with images derived from real-world patients with tables and figures. Finally, we conclude the paper and briefly discuss future work.

## Related works

### Medical image registration techniques

Classic medical image registration methods can be roughly categorized as either feature-based or intensity-based approaches [34]. Feature-based methods represent features with the famous scale-invariant feature transform algorithm (SIFT) [35], which obtains a geometric transformation by extracting corresponding features such as points, lines, vectors, surfaces, and volumes. To a certain extent, these features can reduce the complexity of the registration problem. However, the performance of this type of algorithm mainly depends on its feature design, and the effectiveness of the selected features directly affects the subsequent registration results. Usually, features with conspicuous characteristics are required, and the design of image features with strong expressive abilities remains a problem in registration tasks. On the other hand, for specific image data, finely designed artificial features may provide satisfactory registration results, but there is no guarantee that these features will be well-suited for other data registration tasks.

Regarding intensity-based methods, their process consisting of similarity measurement and optimization has become the major approach for medical image registration due to its high registration accuracy and lack of preprocessing requirements [36]. Generally, pairwise intensity-based medical image registration methods focus on optimizing image similarity, which is a metric that indicates how well images intensities correspond. The goal of pairwise registration is to find transformation parameters that bring two images into correspondence based on their contents. Typically, this process is solved by iteratively optimizing a predefined handcrafted intensity-based dissimilarity metric over the transformation parameters.

Recently, deep learning-based methods have been widely used in medical image registration [37, 38]. These approaches can be simply divided into three categories including deep similarity metrics and supervised and unsupervised transformation estimation methods [39]. Data-driven methods can effectively complete the registration process in a very short time after training them through a suitable framework. However, this idea is often based on large amounts of data labeling and preprocessing, which imposes high requirements on the training data and causes the training process to take a long time. In addition, each trained model usually cannot be effectively transplanted to other datasets. For deep iterative frameworks, an application-specific similarity metric that is learned from architecture neural networks like convolutional neural networks (CNNs) is needed [40], which indicates that an optimization algorithm is also necessary during that process. Other nonparametric approaches tend to be roughly preregistered, while some utilize a multistep process by refining the affine transformation parameters to predict large, global displacements and rotations [41]. It is worth noting that most deep learning-based methods focus on deformable registration rather than linear registration. Compared to commercial software, researchers tend to use some open-source tools such as ANTS [27], Elastix [28] and FSL [29] to accomplish linear as well as coarse registration. In this work, the three commonly used preregistered methods mentioned above are also included in the comparison.

**Fig. 1:**
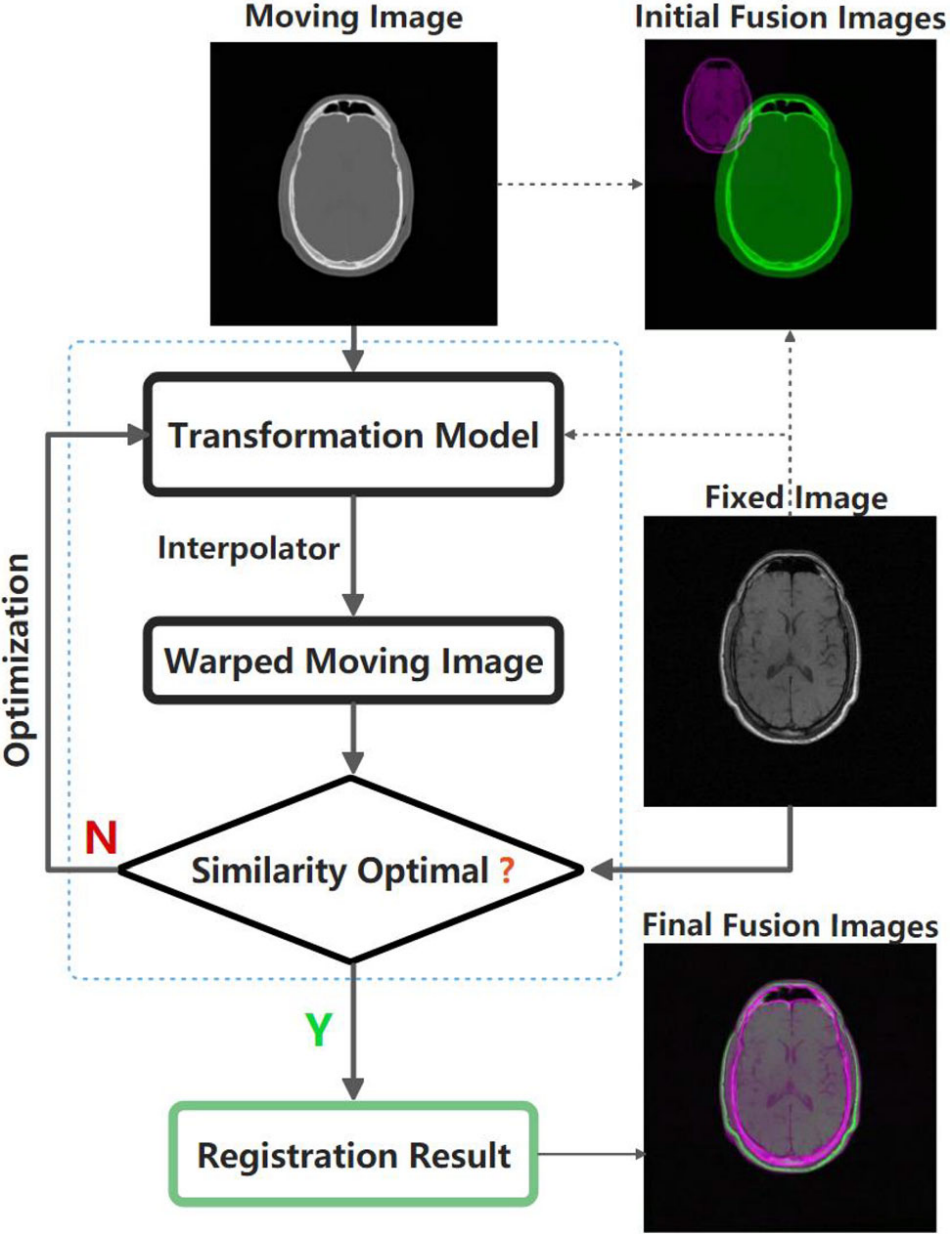
The flowchart of the registration process. The main registration process involves finding a transformation that maximizes or minimizes the similarity metrics between the warped moving image and the corresponding fixed image

### Intensity-based medical image registration algorithms

Intensity-based image registration is a crucial process that involves aligning two or more images by analyzing the intensity values of their pixels. The process is iterative and consists of four key components, including a similarity metric, a parameterizable transformation, an interpolation approach, and an optimization strategy, as illustrated in Fig. 1.

As medical image registration is an optimization problem, an appropriate optimization algorithm is essential for obtaining superior transformation parameters. However, many metaheuristic algorithms that perform well in unconstrained optimization problems may not be suitable for real problems due to the complexity of such problems. For example, the similarity measure employed for unregistered images is usually nonlinear and has numerous local extrema, making it difficult to find the globally best solution. Therefore, an appropriate optimization algorithm can benefit the registration process by finding the best-fitting transformation parameters between the unregistered images in a fast and reliable manner. To address this issue, high-performance algorithms are needed to satisfy the high requirements of intensity-based image registration.

**Table 1:** Comparison of multimodal registration methods adopting metaheuristic algorithms

Method	Algorithm	Modality	Transformation model	Similarity measure	Applied images	Registration tools competition	Outperformed algorithms	Major limitations
[42]	CRO-SL	CT/MRI	Rigid/affine	NMI	Brain	Yes	1. Genetic Algorithm(GA) 2. Scatter Search(SS) 3. Coral Reef Optimization(CRO)	Outdated comparisons algorithms
[43]	DA	CT/MRI	Rigid/ Similarity	NMI	Brain	No	1. Particle Swarm Optimization (PSO) 2. Artificial Bee Colony (ABC)	Tme-consuming,insufficient comparison experiment
[44]	GWO	CT/MRI	Rigid	NMI	Brain	No	1. Particle Swarm Optimization (PSO) 2.Sine Cosine Algorithm (SCA)	Experimental sample size is quite small
[45]	BBO-EL	CT/MRI	Rigid	NMI	Brain	No	1. Biogeography-Based 2. Optimization(BBO) 3. Scatter Search(SS) 4.Coral Reef Optimization(CRO) 5. CRO-SL	Tme-consuming
[46]	HDSA	MRI/MRI	Rigid	CCRE	Brain	No	1. Differential Evolution With Optional External Archive(JADE) 2. Differential Search Algorithm (DSA)	Artificial registration scenarios
[47]	UEO	CT/MRI	Rigid	CCRE	Brain	No	1. Biogeography-Based Optimization(BBO) 2. Gaussian Quantum Behaved Particle Swarm Optimization (GQPSO) 3. Equilibrium Optimizer (EO) 4. HDSA	Lack of comparison to classical registration tools
Ours	NVSA	CT/MRI	Similarity /affine	NMI	Brain	Yes	BSD,BBO-EL,DA,GWO,UEO	

Recently, with the development of multimodal medical image registration, various methods that use metaheuristic algorithms for optimization purposes have been proposed. As shown in Table 1, optimization algorithms including the coral reef optimization algorithm with substrate layers (CRO-SL) [42], the dragonfly algorithm (DA) [43], the gray wolf optimizer (GWO) [44], biography-based optimization with elite learning (BBOEL), the hybrid differential search algorithm (HDSA) [46] and the united equilibrium optimizer (UEO) [47] are applied to intensity-based medical image registration. These algorithms have demonstrated their superiority over many of the other algorithms listed above. However, these methods have certain limitations that restrict their applicability to general multimodal brain tasks. For example, some of these algorithms are based on rigid transformations [44–47] and do not consider affine transformations, which are indispensable in practical applications. In addition, some algorithms are time-consuming [43, 45] and have not been compared with classic registration tools [43–45, 45–47], which limits their practicality.

As seen in Table 1, the normalized mutual information (NMI) [48] is a commonly used measure for calculating global information intensities; other similarity measures such as the cross-cumulative residual entropy (CCRE) [49] have also been proposed. In general, similarity measures such as the NMI and CCRE provide global evaluations of registration quality, while other measures such as normalized cross-correlation (NCC) and the root mean square error (RMSE) [50] may be more localized. Choosing a similarity measure such as the NMI that tends to be global can improve the stability of the utilized algorithm during its iterative process; however, this comes at the cost of ignoring the relevance of some detail areas. On the contrary, if a similarity measure that tends to be local is used as the optimization function, the resulting registration performance may be overestimated despite poor actual alignment results. For example, a high NCC value may not necessarily indicate superior registration performance.

To evaluate the algorithm proposed in this paper more fairly, we use the NMI as the input objective function as well as the global maximum similarity measure as the output function. In addition, the RMSE is chosen as an auxiliary similarity measure for more comprehensive evaluating the registration results. This approach ensures a better overall registration effect and proves the quality of the obtained registration results from the side. The NMI and RMSE of a moving image M and a fixed image F are defined as Eqs. (1) and (2), respectively:

1 $$\begin{aligned} \mathrm{NMI(M,F)}=\frac{H(M)+H(F)}{2H(M,F)} \end{aligned}$$

Here *H* indicates Shannon entropy.

2 $$\begin{aligned} \textrm{RMSE} = \sqrt{\frac{1}{n} \sum _{i=1}^n(M_{i}-{F}_{i})^2} \end{aligned}$$

Here *n* indicates the number of image pixels.

## Proposed method

### BSD

BSD [22] was inspired by Bernstein polynomials; it is considered a new universal differential evolution (uDE) algorithm due to its lack of internal control parameters. BSD is described as an easily controllable, simple-structured, nonrecursive, highly efficient, fast, and practically parameter-free uDE algorithm. In addition, the BSD proved its superiority over other algorithms such as the ABC algorithm [23], the JADE algorithm [24], CUCKOO [25], and the WDE algorithm [26] in a comparison concerning numerical function optimization and image evolution problems. The essential parts of the BSD algorithm are summarized as follows, and the relevant nomenclature is summarized in Table 2:

**Table 2:** Nomenclature

Symbol	Meaning/Definition
$f(\cdot )$	Objective function
fitP	Function evaluation values
low,up	Lower and upper limits of search-space
*N*	Size of pattern matrix
*D*	Dimension of problem
Epoch	Maximum number of iterations
$best_{sol}$	Global minimum value
$best_{PV}$	The global minimizer pattern vector
$k(\cdot ) \sim {\textbf {U}} (0,1)$	*k* is a uniform random number
$\lambda (\cdot ) \sim {\textbf {N}} (0,1)$	$\lambda$ is a normal random number
$\eta (\cdot ) \sim {\textbf {N}} (0,1)$	$\eta$ is a normal random number
$\beta (\cdot ) \sim {\textbf {U}} (0,1)$	$\beta$ is a uniform random number
${P}_{i,j} | {P}_{i,j} \sim {\textbf {U}} (low_j,up_j)$	Pattern vectors of pattern matrix
*Permute()*	Permuting function
*M*	Activation matrix

#### Initialization and function evaluation

Similar to other uDE algorithms, in BSD, *N* individuals are randomly generated in a *D*-dimensional search space within the certain boundary range (*up* and *low*) using Eq. (3):

3 $$\begin{aligned} {P}_{i,j}^{\rm{Initial}}={\rm{low}}_{j}+\alpha \cdot ({\rm{up}}_{j}-{\rm{low}}_{j}) \ | \ \alpha \sim {\textbf {U}} (0,1) \qquad {i\in [1:N];j\in [1:D]} \end{aligned}$$

Then, the function evaluation values *fitP* are calculated based on the objective function *f* using Eq. (4):

4 $$\begin{aligned} {fitP}_{i}=f({P}_{i}) \qquad {i\in [1:N]}, \end{aligned}$$

#### Setting the best pattern vector

The best pattern vectors obtained thus far ${best}_{PV}$ are defined as Eq. (5), and the objective function value calculated with this set of vectors ${best}_{sol}$ is regarded as the global best vector and produces the best fitness value.

5 $$\begin{aligned}{}[{best}_{sol},{best}_{PV}] = [{fitP}_{(\gamma )},{P}_{(\gamma )}] \ | \ {fitP}_{(\gamma )}=min(fitP) \qquad {\gamma \in [1:N]} \end{aligned}$$

#### Crossover ratio

**Fig. 2:**
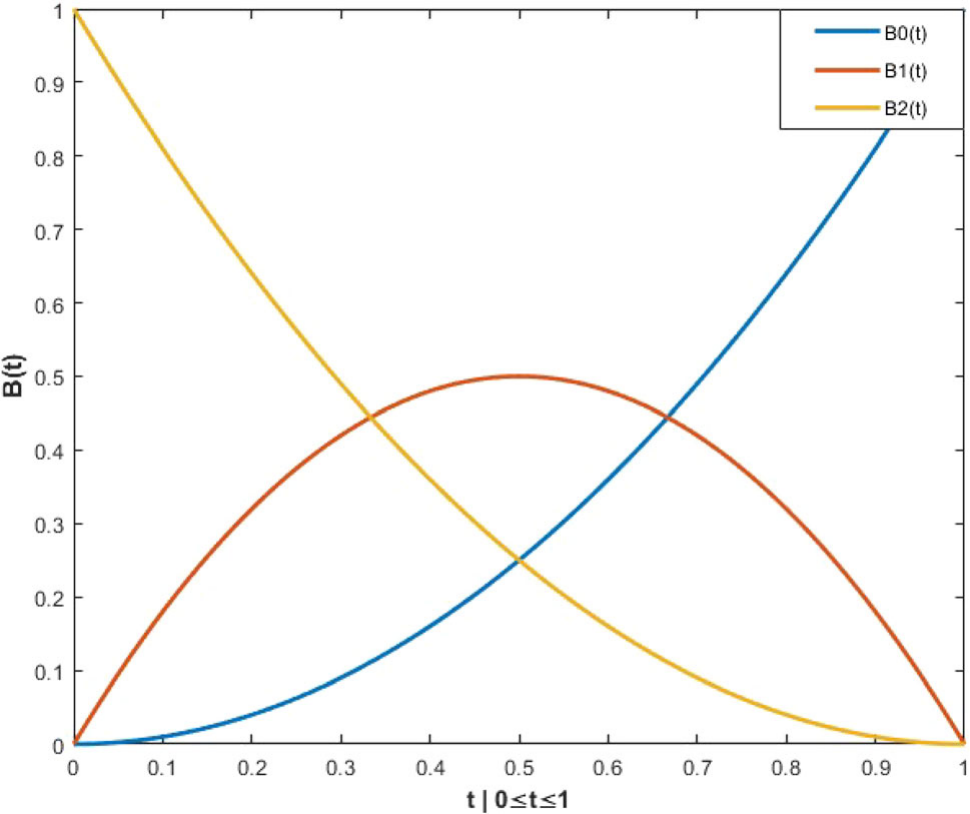
$2^{nd}$ degree Bernstein polynomials

BSD manipulates the crossover [51, 52] ratio with an activation matrix *M*. The initial matrix *M* is a zero matrix. For every individual *i*, *M* is determined by using Eq. (6):

6 $$\begin{aligned} {M}_{i,u(1:[\rho .D])}=1 \ | \ u = Permute([1:D]) \end{aligned}$$

where $permute(\cdot )$ is a sorter that rearranges the order of the $elements(\cdot )$. $\rho$ is defined using Eq. (7);

7 $$\begin{aligned} \rho = {\left\{ \begin{array}{ll} (1-\beta )^2 &{} k=1 \\ 2\beta \cdot (1-\beta ) &{} k=2 \\ \beta ^2 &{} k=3 \end{array}\right. } \end{aligned}$$

where $\beta \sim {\textbf {U}} (0,1)$, and $k\in [1:3]$. The value of $\rho$ can be calculated with $2^{nd}$-degree Bernstein polynomials. Specifically, $\beta$ is given to one of three cases that obey $2^{nd}$-degree Bernstein polynomials. As shown in Fig. 2, a $\beta$ generated in the range of [0,1] is given to one of three cases that obey $2^{nd}$-degree Bernstein polynomials.

#### Search range of the population

The BSD algorithm computes the search range of the population using Eq. (8). Here, the parameters $\eta \sim {\textbf {U}} (0,1)$ and $\lambda \sim {\textbf {N}} (0,1)$ represent uniform and normal distributions, respectively. *u* and *v* are dynamic selectors whose default settings are $u\sim {\textbf {U}} (0,1)$ and $v\sim {\textbf {U}} (0,1)$, respectively. Furthermore, a $(\cdot ,\cdot )$ sized all-ones matrix is defined as $Q(\cdot ,\cdot )$ = 1.

8 $$\begin{aligned} F= {\left\{ \begin{array}{ll} ([\eta _{(1,1:D)}^3\ \times |\lambda _{(1,1:D)}^3|]' \cdot Q_{(1,1:N)})' &{} u<v \\ \lambda _{(N,1)}^3\ {\times Q}_{(1,D)} &{} u\ge {v} \end{array}\right. } \end{aligned}$$

#### Pattern vectors

For the $i-th$ individual among the best pattern vectors obtained thus far ${best}_{PV}$ and the current pattern vectors *P*, the trial pattern vector *T* is executed to evaluate the new objective function value.

9 $$\begin{aligned} T= & {} P+F\cdot M\cdot E \cdot {(W^*)}^3+(1-{(W^*)}^3) \cdot {best}_{PV}\nonumber \\{} & {} \quad -P \ | \ {W^*}_{(1:N,1)} \sim {\textbf {U}} (0,1) \end{aligned}$$

Where $E=W \cdot {P}_{L1}+(1-W)\cdot {P}_{L2} \ | \ {W}_{1:N,1:D}\sim {\textbf {U}} (0,1)$, and *L*1, *L*2 are defined in Eq. (10)

10 $$\begin{aligned} L1=\textrm{Permute}\left( 1:N\right) ,\ L2=\textrm{Permute}\left( 1:N\right) \ | \ L1\ne \left[ 1:N\right] ,L1\ne L2 \end{aligned}$$

#### Boundary control

Once the candidate of a trial pattern vector exceeds the preset search space, it is randomly generated in the search space based on the boundary control strategy defined in Eq. (11).

11 $$\begin{aligned} {T}_{i,j}=\textrm{low}_j+{\alpha } \cdot (up_j-{low}_{j})\ |\ T_{i,j} < \textrm{low}_j \ or\ T_{i,j} > up_j\ | \ \alpha \sim {\textbf {U}} (0,1) \end{aligned}$$

### NVSA

In this section, the motivation and working principles of the NVSA are introduced; then, the efficiency of the NVSA is discussed. We improve the NVSA by replacing the $2^{nd}$-degree Bernstein polynomials with the normal vibration distribution. This vibration can be run by default or fine-tuned by some control parameters. Correspondingly, we redesign the update method for the trial pattern vector and adjusting it with the search strategy.

#### Normal vibration distribution

The BSD algorithm computes the crossover ratio with 2nd-degree Bernstein polynomials. To be specific, every individual randomly chooses a value from zero to one, which is computed by one of three Bernstein polynomials functions using Eq. (7). As Fig. 2 illustrates, most of the *B*(*t*) values tend to be located in a small area. For example, given a random *t* value, there is a 77.8% probability that the number of population activations *B*(*t*) is less than or equal to 0.5. which represents the fact that most of the best-so-far candidates still remain in each iteration. Notably, this working principle favors exploitation over exploration.

To better balance the exploration and exploitation abilities of the developed algorithm, we initially try to replace the Bernstein polynomial functions with the normal distribution. However, a simple normal distribution does not successfully meet our verification standards due to the fact that a single normal distribution is unable to satisfy the crossover property as well as the mutation needs of the algorithm. Thus, a vibration function is added to the normal distribution to increase its population diversity. Finally, to strengthen the robustness of the proposed method when facing various real-world problems, we also set some control parameters to control the vibration process. Here, the NVSA proposes a new method to obtain the activation matrix M using Eq. (12) and applies Eq. (13) to replace Eq. (7) in the BSD algorithm.

12 $$\begin{aligned} \rho =\; & {} \root G \of {exp} + 2k_1 (1-k_1)- H \end{aligned}$$

13 $$\begin{aligned} H =\; & {} \textrm{exp}^{(\frac{1}{G(k_4-k_5)^2+P})} \ | \ G=a+(b-a)k_3; P= c+(d-c)k_2 \end{aligned}$$

where exp indicates natural constant *e* and $k_{1-5}\sim {\textbf {U}} (0,1)$ denotes the uniform distribution. *a*, *b*, *c*, and *d* are parameters for controlling the vibration, and their default values are set as 1000, 10,000, 3, and 5, respectively. As a result, the values of *G* and *P* are decided by the above four parameters, where *G* and *P* are randomly generated between [1000, 10,000] and [3,5], respectively. It is worth noting that the four parameters mentioned above can be manually set when facing various optimization problems.

**Fig. 3:**
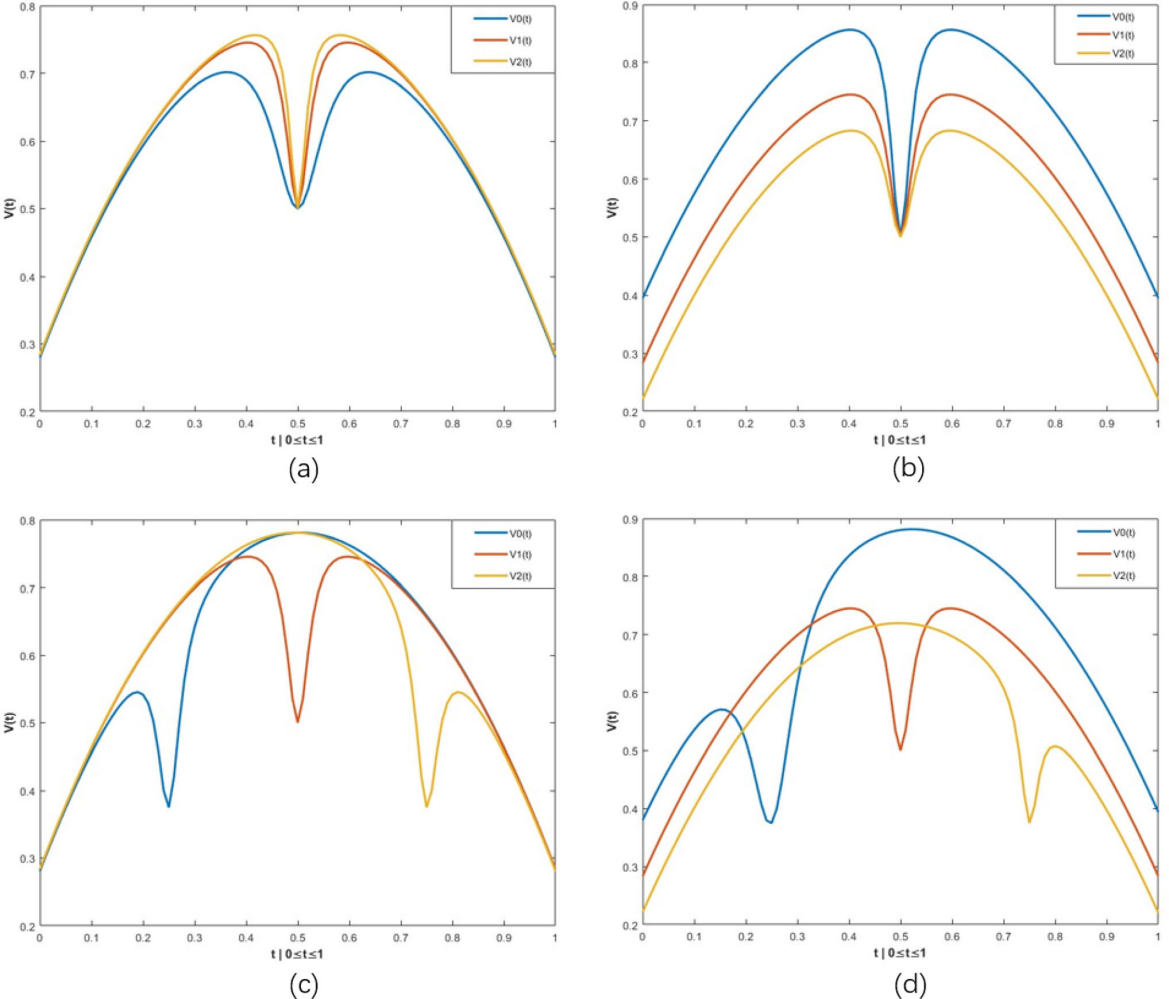
Four kinds of random parameters setting illustration, the specific parameter settings are referred to Table 1

For ease of understanding, Table 3 and Fig. 3 illustrate different kinds of vibration with normal distributions. To be specific, we give each parameter *G*, *P*, and *k*5 three different values in Table 3, along with the corresponding graph shown in Fig. 3, to help the reader better understand how the vibration function works. For example, *V*0(*t*), *V*1(*t*), and *V*2(*t*) in Fig. 3a utilizes three different *G* values (1000, 5000, 10,000), while $P=4$ and $k5=0.5$ remain unchanged.

It can be seen that *G* controls the vibration width. The larger the value of *G* is, the narrower the width of the vibration (see Fig. 3a). *P* controls the maximum value of the entire normal distribution. The smaller the value of *P* is, the larger height of the entire distribution (see Fig. 3b). Additionally, *k*5 decides where the vibration appears. The locations of vibrations tend to be generated from left to right as the value of *k*5 increases from zero to one (see Fig. 3c). In short, a vibration can be generated at any point of the unique normal distribution with a random vibration width (see Fig. 3d).

Compared to the $\rho$ obtained using 2nd-degree Bernstein polynomials in Eq. (7), this novel normal vibration distribution (1) increases the probability of the overall crossover ratio, which is helpful for exploration in the early stage; (2) replaces the constant 2nd-degree Bernstein polynomials with a dynamic normal distribution that changes over time, which helps promote high algorithmic diversity; and (3) makes the algorithm more variable than the ordinary normal distribution with the addition of vibration.

**Table 3:** Different value indicates to Fig. 3

Parameter	Figure	Fig. 3a	Fig. 3b	Fig. 3c	Fig. 3d
G	V0(t)	1000	5000	5000	1000
	V1(t)	5000			5000
	V2(t)	10,000			10,000
P	V0(t)	4	3	4	3
	V1(t)		4		4
	V2(t)		5		5
k5	V0(t)	0.5	0.5	0.25	0.25
	V1(t)			0.5	0.5
	V2(t)			0.75	0.75

#### Search strategy

In this section, we propose a new search pattern to cooperate with the normal vibration distribution. This is because the search strategy of the original BSD algorithm may not suit the proposed method.

That is, the NVSA uses a smaller search step size to match the high-frequency crossover probability. Another reason for this is that the NVSA is presented to solve medical image registration problems, and this requires us to make corresponding improvements to fit the task needs. To be specific, two places are modified. First, we adjust the search range of the population using Eq. (14).

14 $$\begin{aligned} F= {\left\{ \begin{array}{ll} \eta _{(1:N,1:D)}^3 &{} u^3<v \\ \lambda _{(1:N,1:D)} &{} u^3\ge {v} \end{array}\right. } \end{aligned}$$

As in Eq. (8), the parameters $\eta \sim {\textbf {U}} (0,1)$, $v\sim {\textbf {U}} (0,1)$, and $u\sim {\textbf {U}} (0,1)$ here are for the uniform distribution, while $\lambda \sim {\textbf {N}} (0,1)$ is used for the normal distribution.

Compared to Eq. (8), these modifications simplify the generation of *F* and increase the search step size of the population to enhance the exploitation ability of the algorithm. This improvement makes the NVSA tend toward active iteration with smaller search steps, and the algorithm can be steadily updated during the early stage of the iterative process. It is easier to find the global optimal result in the later stage of iteration. By combining this improvement with the normal vibration distribution, the NVSA can better enhance the exploration capabilities of the algorithm in the early stage and its exploitation ability in the later stage.

Second, we enhance the variability of Eq. (9) by replacing the associated matrixes with independent versions using Eq. (15).

15 $$\begin{aligned} T =P+M\cdot F \cdot (s \cdot (w_1 \cdot P_{L1})+w_2 \cdot P_{L2})+w_3 \cdot {\rm{best}}_{\rm{PV}}-P) \end{aligned}$$

where *L*1 and *L*2 remain unchanged compared to Eq. (10). ${s}_{(1:N,1:D)} \sim {\textbf {N}} (0,1)$ signifies the normal distribution, while $w_1$, $w_2$ and $w_3$ represents three different kinds of uniform distributions; ${w}_{1-3,(1:N,1:D)}\sim {\textbf {U}} (0,1)$.

Two aspects motivate this modification. First, we notice that $W^*$ and $(1-W^*)$ interfere with each other in Eq. (9), which may decrease the diversity of the population. Second, ${W^*}_{(1:N)}\sim {\textbf {U}} (0,1)$ only provides a single function for restricting the search range. Hence, we make two changes: first, we replace *W*, $(1-W)$, $W^*$, and $(1-W^*)$ in Eq. (9) with four independent parameters *s*, *w*1, *w*2 and *w*3, respectively. These unrelated parameters can enhance the diversity of the population.

The second change is to replace ${W^*}_{(1:N)}\sim {\textbf {U}} (0,1)$ with new parameters ${s}_{(1:N,1:D)}\sim {\textbf {N}} (0,1)$. In this *s*, *D* stands for the number of optimization problems under consideration, which makes *s* a matrix rather than a vector. As *s* obeys a normal distribution, not only can *s* reduce the step size but it also provides the ability to enlarge the step size. In addition, *s* also effects the search direction of the algorithm, which supplements the search orientation in the first case of Eq. (9).

Finally, compared to *F* and *T* in Eqs. (8) and (9), this modified search strategy (1) adjusts the search range of the population so that the search strategy can be more efficiently matched with the normal vibration distribution; (2) changes the interrelated random parameters into unrelated parameters, which improves the diversity of the algorithm; and (3) uses the normal distribution to expand the selectivity of the search direction of the algorithm so that the algorithm does not easily fall into local optima.

In the end, we propose a brand-new adjustable mutation strategy with a corresponding search structure named the NVSA. These modifiers (1) improve the early-stage global convergence ability of the algorithm; (2) increase the diversity of the algorithm by inventing a new mutation strategy; (3) provide more direction for the search process during the optimization procedure by redesigning the search strategy; and (4) improve the adaptability of the algorithm when facing different optimization problems by adding adjustable control parameters. The pseudocode of the NVSA for medical image registration is shown in Algorithm 1.
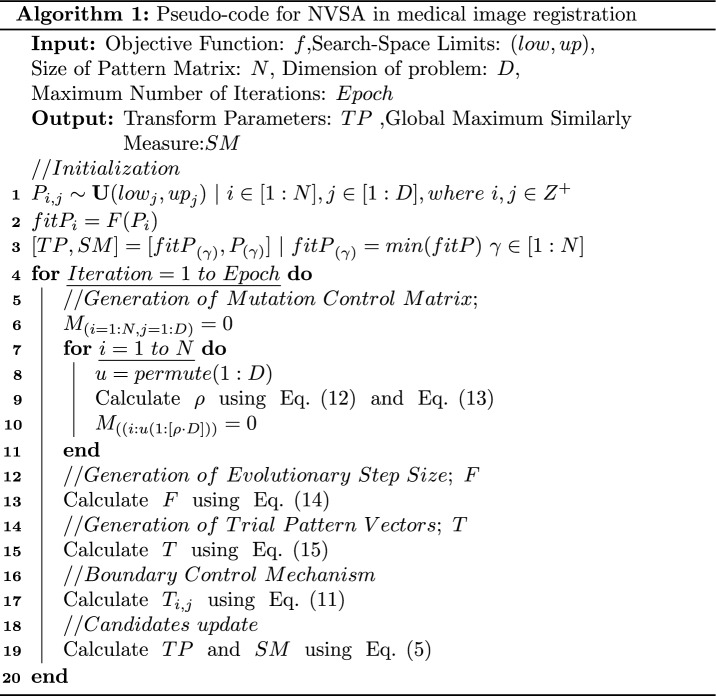


## Results obtained on benchmark functions

In this section, the NVSA is compared to two categories of existing optimization methods in optimization test problems: (1) Bernstein search-based algorithms, including BSD [22] and the Bezier search-based DE algorithm (BeSD) [53]. (2) In recent years, high-performance optimizers, including the Coronavirus herd immunity optimizer (CHIO) [54], Archimedes optimization algorithm (archOA) [55], and Bernstein-Levy DE algorithm (BDE) [56], have also been developed.

Reference [22] demonstrated that BSD can outperform the ABC [23], JADE [24], CUCKOO [57] and WDE [26] algorithms. Furthermore, the BeSD [53] and BDE [56] algorithms are both modified versions of BSD, sharing similar structures. These methods have proven their superiority over the covariance matrix learning and searching preference (CRMLSP) method, the mean-variance optimization algorithm (MVO), without approximation optimization (WA), SHADE and LSHADE in solving numerical problems. The most relevant provisions are as follows.

- PlatEMO [58] is adopted as the optimization platform, and the default parameters of each algorithm are used.
- The algorithms use 50 particles along with 1000 iterations with 30 independent runs (the total numbers of cost function evaluations is 1,50,000).
- We use 23 well-known classic benchmark functions [58].
- The mean value and mean standard deviation (STD) are used as performance indicators.
- A two-tailed Wilcoxon signed rank test was used for the statistical comparison of the results obtained from the experiments. In statistical comparisons, the level of significance is set to 0.05.
- Testing is performed using MATLAB R2022a on a Windows 10 operating system with an Intel (R) Core (TM) i7-8550U CPU @ 1.80 GHz$-$2.00 GHz and 16.00 GB of DDR4 RAM.

**Table 4:** Comparative results by using 50 particles on classic benchmark functions,The bests are highlighted in bold

Problem	archOA	BDE	BeSD	BSD	CHIO	NVSA
SOP_F1	4.7517e+1 (3.59e+1) -	1.7614e$-$1 (1.79e$-$1) -	3.2540e+3 (1.71e+3) -	3.2639e$-$10 (1.13e$-$9) -	5.1774e+4 (6.07e+3) -	**9.2834e** $-$**103 (3.27e** $-$**102)**
SOP_F2	1.9112e+0 (6.90e$-$1) -	2.3345e+0 (6.15e$-$1) -	1.7307e+5 (8.95e+5) -	3.1545e$-$3 (1.26e$-$3) -	8.7532e+8 (2.28e+9) -	**3.8418e** $-$**26 (1.09e** $-$**25)**
SOP_F3	2.1997e+3 (5.29e+3) -	9.1530e+7 (1.53e+7) -	8.3843e+7 (1.96e+7) -	9.4550e+7 (0.00e+0) -	7.2445e+4 (1.31e+4) -	**4.2525e** $-$**3 (2.13e** $-$**2)**
SOP_F4	4.3698e+0 (4.38e+0) -	1.7478e+0 (4.79e$-$1) -	8.1946e$-$1 (1.28e+0) -	6.1314e$-$1 (2.14e$-$1) -	8.2489e+1 (4.52e+0) -	**2.2896e** $-$**17 (2.60e** $-$**17)**
SOP_F5	**1.0581e+3 (1.51e+3) +**	2.1270e+9 (2.15e+8) -	2.1950e+9 (0.00e+0) -	1.8896e+9 (3.71e+8) -	1.6370e+8 (3.28e+7) +	1.4282e+9 (2.45e+7)
SOP_F6	4.9400e+1 (3.88e+1) -	0.0000e+0 (0.00e+0) =	7.3720e+3 (3.80e+3) -	0.0000e+0 (0.00e+0) =	5.1971e+4 (5.91e+3) -	**0.0000e+0 (0.00e+0)**
SOP_F7	**4.4811e** $-$**2 (2.05e** $-$**2) +**	4.8096e$-$1 (2.70e$-$1) =	4.9415e$-$1 (2.92e$-$1) =	4.8717e$-$1 (2.84e$-$1) =	7.8901e+1 (1.88e+1) -	5.5817e$-$1 (2.55e$-$1)
SOP_F8	− 3.0187e+3 (5.41e+2) -	− 5.4177e+3 (1.85e$-$12) =	− 5.4177e+3 (1.85e$-$12) =	− 5.4177e+3 (1.85e$-$12) =	− 3.6861e+3 (3.62e+2) -	**− 5.4177e+3 (1.85e** $-$**12)**
SOP_F9	8.9311e+1 (7.96e+1) -	8.6774e+2 (3.47e$-$13) -	8.6774e+2 (3.47e$-$13) -	8.6774e+2 (3.47e$-$13) -	3.7252e+2 (1.66e+1) -	**0.0000e+0 (0.00e+0)**
SOP_F10	1.8775e+1 (4.54e+0) -	6.6365e$-$1 (3.26e$-$1) -	7.7177e+0 (1.11e+0) -	1.1810e$-$3 (4.19e$-$4) -	2.0151e+1 (2.71e$-$1) -	**1.8208e** $-$**14 (7.23e** $-$**15)**
SOP_F11	1.2765e+0 (1.61e$-$1) -	9.4997e$-$2 (1.05e$-$1) -	9.3617e$-$1 (1.21e$-$2) -	4.3634e$-$5 (2.38e$-$4) -	4.6765e+2 (4.36e+1) -	**0.0000e+0 (0.00e+0)**
SOP_F12	2.7324e+1 (2.61e+1) -	3.3763e+0 (6.29e$-$1) +	2.6675e+2 (2.55e+2) -	**3.1415e+0 (1.51e** $-$**4) +**	3.2963e+8 (1.07e+8) -	9.4772e+0 (2.72e+0)
SOP_F13	5.7860e+0 (2.89e+0) -	2.9769e+0 (9.43e$-$3) -	**6.3792e** $-$**1 (5.91e** $-$**1) +**	3.0000e+0 (5.57e$-$6) -	6.7573e+8 (1.49e+8) -	1.6303e+0 (6.09e$-$1)
SOP_F14	**4.2352e+0 (3.93e+0) +**	1.4541e+1 (1.81e$-$15) +	1.4541e+1 (2.32e$-$9) -	1.4541e+1 (1.89e$-$15) +	1.7402e+1 (1.84e+1) =	1.4541e+1 (1.08e$-$11)
SOP_F15	4.8295e$-$1 (1.68e+0) -	1.4778e$-$1 (1.07e$-$8) -	1.4763e$-$1 (1.08e$-$4) +	1.4762e$-$1 (1.37e$-$4) +	**6.4129e** $-$**2 (5.42e** $-$**2) +**	1.4775e$-$1 (5.26e$-$5)
SOP_F16	**− 1.0307e+0 (2.03e** $-$**3) +**	3.6181e+0 (1.30e$-$15) -	3.6181e+0 (1.45e$-$7) +	3.6181e+0 (1.48e$-$15) -	− 3.3934e$-$1 (7.05e$-$1) +	3.6181e+0 (3.54e$-$9)
SOP_F17	**4.1032e** $-$**1 (1.67e** $-$**2) +**	4.3745e+1 (7.23e$-$15) -	4.3745e+1 (2.84e$-$8) -	4.3745e+1 (3.00e$-$14) -	1.0656e+0 (4.53e$-$1) +	4.3745e+1 (2.54e$-$7)
SOP_F18	**4.0566e+0 (4.91e+0) +**	7.6728e+4 (0.00e+0) =	7.6728e+4 (0.00e+0) =	7.6728e+4 (0.00e+0) =	2.1050e+1 (1.65e+1) +	7.6728e+4 (0.00e+0)
SOP_F19	**− 3.8020e+0 (5.59e** $-$**2) +**	− 6.7974e$-$2 (4.23e$-$17) =	− 6.7974e$-$2 (4.23e$-$17) =	− 6.7974e$-$2 (4.23e$-$17) =	− 3.6098e+0 (2.18e$-$1) +	− 6.7974e$-$2 (4.23e$-$17)
SOP_F20	**− 2.7873e+0 (3.06e** $-$**1) +**	− 5.1072e$-$3 (0.00e+0) =	− 5.1072e$-$3 (0.00e+0) =	− 5.1072e$-$3 (0.00e+0) =	− 2.1979e+0 (4.14e$-$1) +	− 5.1072e$-$3 (0.00e+0)
SOP_F21	**− 3.4327e+0 (2.21e+0) +**	− 2.7312e$-$1 (1.13e$-$16) =	− 2.7312e$-$1 (1.13e$-$16) =	− 2.7312e$-$1 (1.13e$-$16) =	− 8.8021e$-$1 (4.33e$-$1) +	− 2.7312e$-$1 (1.13e$-$16)
SOP_F22	**− 3.6981e+0 (2.23e+0) +**	− 2.9362e$-$1 (5.65e$-$17) =	− 2.9362e$-$1 (5.65e$-$17) =	− 2.9362e$-$1 (5.65e$-$17) =	− 1.2488e+0 (5.67e$-$1) +	− 2.9362e$-$1 (5.65e$-$17)
SOP_F23	**− 2.7243e+0 (1.14e+0) +**	− 3.2173e$-$1 (0.00e+0) =	− 3.2173e$-$1 (0.00e+0) =	− 3.2173e$-$1 (0.00e+0) =	− 1.3275e+0 (4.90e$-$1) +	− 3.2173e$-$1 (0.00e+0)

**Table 5:** Comparison of classic benchmark problems solving successes of NVSA and tested methods by using Wilcoxon signed rank test (p = 0.05)

Problem	archOA				BDE				BeSD				BSD				CHIO			
	p	R+	R-	stat	p	R+	R-	stat	p	R+	R-	stat	*p*	R+	R-	stat	p	R+	R-	stat
SOP_F1	4.11E-07	0	465	−	1.73E-06	0	465	−	1.73E-06	0	465	−	1.73E-06	0	465	−	1.73E-06	0	465	−
SOP_F2	1.73E-06	0	465	−	1.73E-06	0	465	−	1.73E-06	0	465	−	1.73E-06	0	465	−	1.73E-06	0	465	−
SOP_F3	3.52E-06	7	458	−	1.73E-06	465	0	+	1.73E-06	0	465	−	1.92E-06	464	1	+	1.73E-06	0	465	−
SOP_F4	1.73E-06	0	465	−	2.35E-06	3	462	−	2.35E-06	435	30	+	2.35E-06	3	462	−	1.73E-06	0	465	−
SOP_F5	2.13E-06	463	2	+	1.73E-06	465	0	+	1.73E-06	0	465	−	1.73E-06	465	0	+	8.77E-01	240	225	=
SOP_F6	2.77E-03	378	87	+	1.73E-06	0	465	−	1.73E-06	441	24	+	6.32E-05	427	38	+	6.98E-06	451	14	+
SOP_F7	1.73E-06	465	0	+	1.73E-06	0	465	−	1.73E-06	462	3	+	1.73E-06	0	465	−	1.73E-06	465	0	+
SOP_F8	1.73E-06	465	0	+	1.73E-06	0	465	−	1.73E-06	12	453	−	1.73E-06	0	465	−	1.73E-06	465	0	+
SOP_F9	1.73E-06	465	0	+	1	0	0	=	1	0	0	=	1	0	0	=	1.73E-06	465	0	+
SOP_F10	1.73E-06	465	0	+	1	0	0	=	1	0	0	=	1	0	0	=	1.73E-06	465	0	+
SOP_F11	1.73E-06	0	465	−	1.73E-06	0	465	−	1.73E-06	0	465	−	1.73E-06	0	465	−	1.73E-06	0	465	−
SOP_F12	1.73E-06	465	0	+	1	0	0	=	1	0	0	=	1	0	0	=	1.73E-06	465	0	+
SOP_F13	1.73E-06	465	0	+	1	0	0	=	1	0	0	=	1	0	0	=	1.73E-06	465	0	+
SOP_F14	1.73E-06	465	0	+	1	0	0	=	1	0	0	=	1	0	0	=	1.73E-06	465	0	+
SOP_F15	1.73E-06	465	0	+	1	0	0	=	1	0	0	=	1	0	0	=	1.73E-06	465	0	+
SOP_F16	1.73E-06	0	465	−	1.73E-06	0	465	−	1.73E-06	0	465	−	1.73E-06	0	465	−	1.73E-06	0	465	−
SOP_F17	1.73E-06	0	465	−	1.73E-06	0	465	−	1.73E-06	0	465	−	1.73E-06	0	465	−	1.73E-06	0	465	−
SOP_F18	1.73E-06	0	465	−	1.73E-06	0	465	−	1.73E-06	0	465	−	1.73E-06	0	465	−	1.73E-06	0	465	−
SOP_F19	1.73E-06	465	0	+	2.13E-06	2	463	−	2.13E-06	0	465	−	3.11E-05	30	435	−	1.73E-06	465	0	+
SOP_F20	1.73E-06	0	465	−	1	0	0	=	1	0	465	−	1	0	0	=	1.73E-06	0	465	−
SOP_F21	1.73E-06	465	0	+	2.54E-01	288	177	=	2.54E-01	276	189	=	2.80E-01	285	180	=	1.73E-06	0	465	−
SOP_F22	1.73E-06	0	465	−	1	0	0	=	1	0	0	=	1	0	0	=	1.73E-06	0	465	−
SOP_F23	1.73E-06	0	465	−	4.32E-08	0	465	−	4.32E-08	0	465	−	4.32E-08	0	465	−	1.73E-06	0	465	−
±/=				12/11/0				2/12/9				3/12/8				3/11/9				10/12/1

+: tested method is winner, -:VNSA is winner, =: Similar performance

In Table 4, we calculated the mean and standard deviation values (in brackets) for the $SOP_{F1}-SOP_{F23}$ problems obtained by NVSA and the test methods. In Table 5, we present the results of a statistical comparison based on the Wilcoxon signed rank test (p = 0.05) of the numerical problem-solving success of NVSA and the tested methods for $SOP_{F1}-SOP_{F23}$.

In the last rows of Table 5, the results obtained from the NVSA and the other tested methods are compared in terms of (+, −, =). (+) represents that the tested method obtains a statistically better result than that of the NVSA. (−) signifies that the NVSA obtains a statistically better result than the tested method. (=) denotes that the performances of the NVSA and the related tested method are statistically equal. 50 particles along with 1000 iterations and 30 independent runs: archOA (12,11,0), BDE (2, 12, 9), BeSD (3, 12, 8), BSD (3, 11, 9), and CHIO (10, 12, 1).

In summary, the results verify the superior performance of the NVSA over the other 5 metaheuristic algorithms in terms of solving various benchmark functions. These two tables demonstrate that the NVSA is significantly better than all Bernstein search-based algorithms: the BSD, BeSD and BDE algorithms. Moreover, the NVSA is slightly superior to CHIO and is competitive with archOA.

## Multimodal medical image registration results

### Materials

**Fig. 4:**
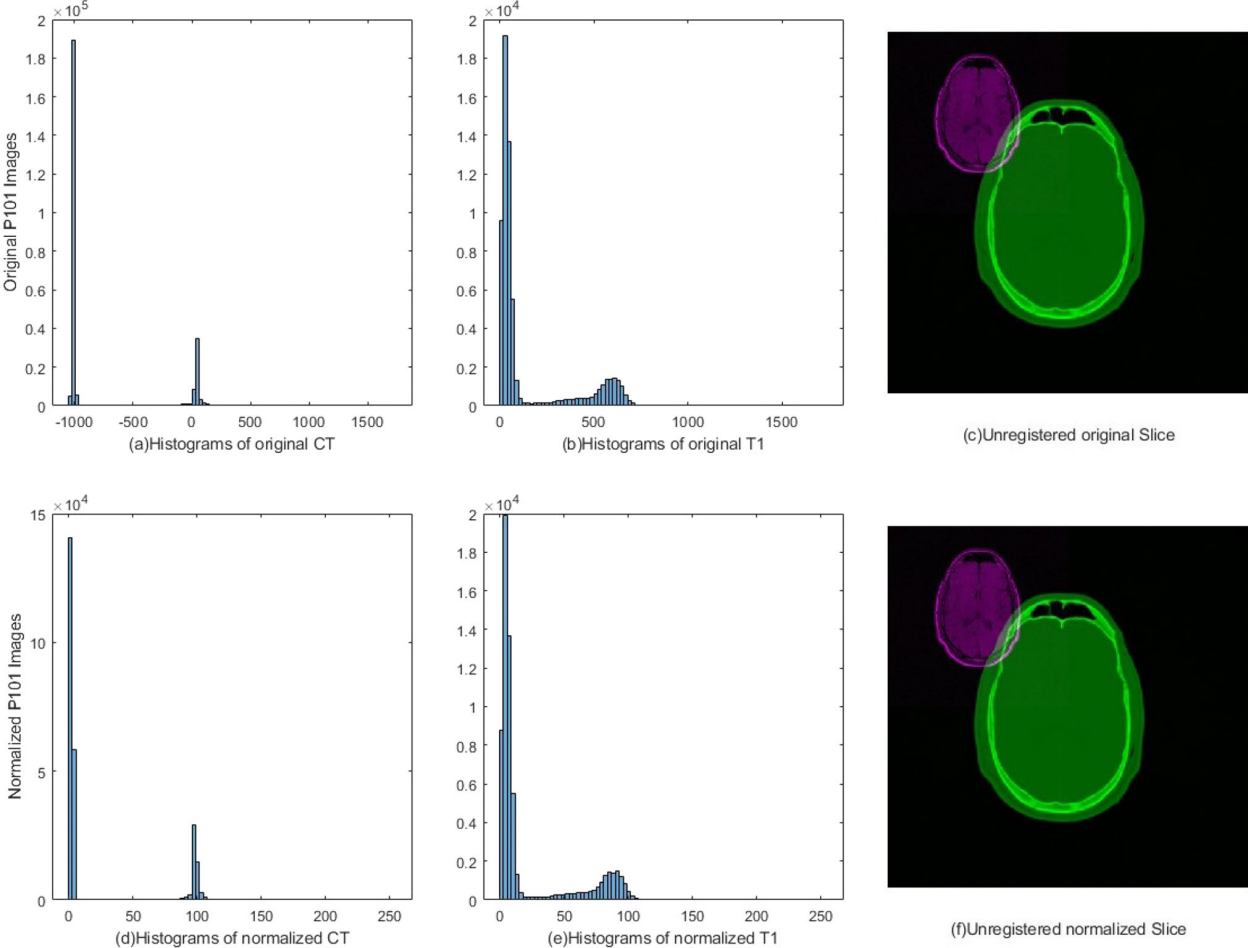
Histograms of Patient001 original images (up) and normalized images (down)

In the following experiments, the Retrospective Image Registration Evaluation (RIRE) database [59] is chosen as the dataset because it is one of the most frequently used datasets for benchmarking the performance achieved by metaheuristic algorithms in multimodal image registration tasks [60]. We take CT images (512 $\times$ 512 $\times$28–34 voxels) as moving images and MR images, including T1, T2, PD images (256 $\times$ 256 $\times$ 20–26 voxels) as fixed images. Furthermore, the pixels of these images are normalized to [0,256] to better measure and accomplish the image registration task. Figure 4 illustrates the original and normalized CT and MR images of patient-001.

**Fig. 5:**
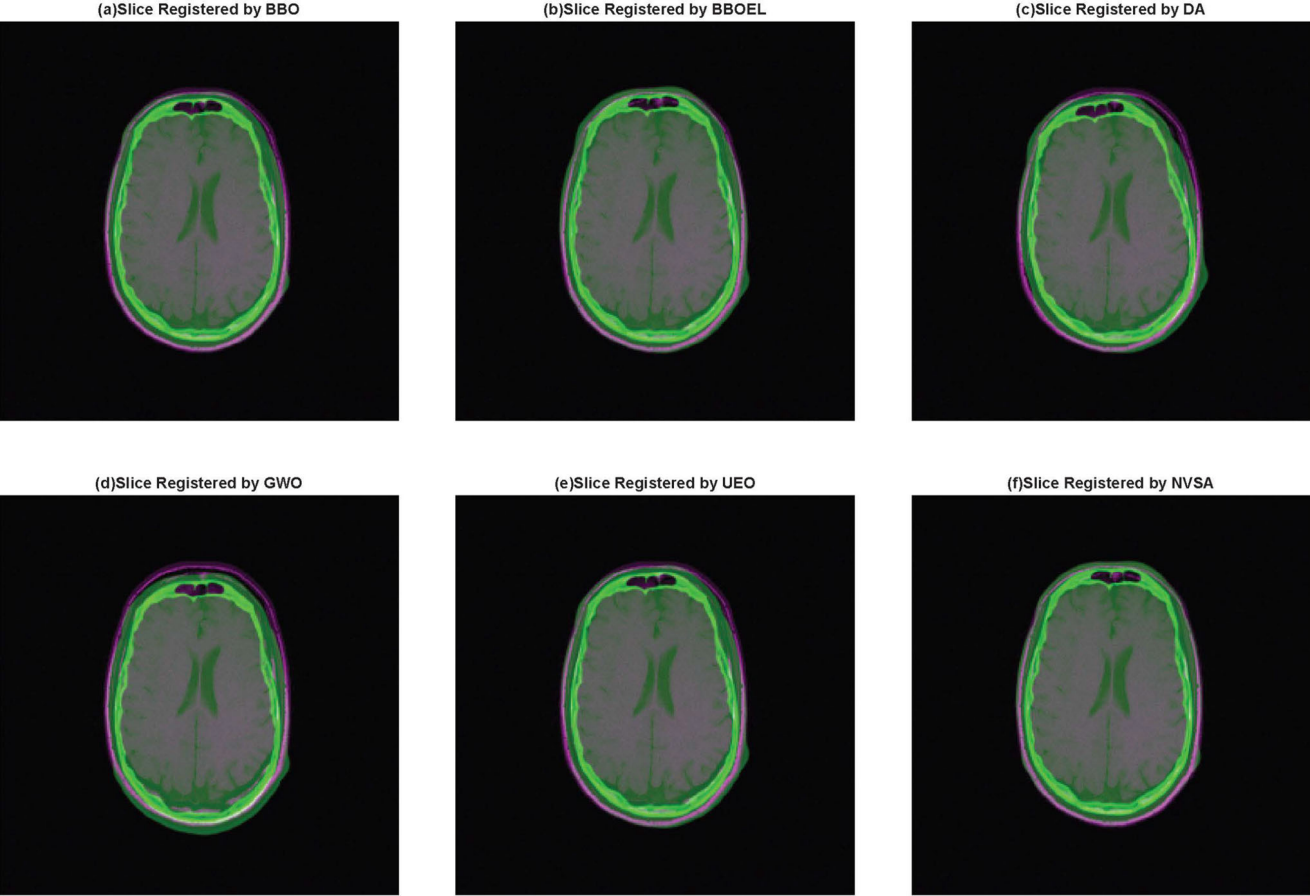
Visualizations of the registration process between multimodal P002 CT-T1 image by using six different metaheuristic algorithms

### Comparisons with SOTA algorithms

In this section, six metaheuristic algorithms, including the proposed NVSA and the original BSD algorithm [22], the high-performance DA [43] and GWO [44] algorithms, and the SOTA BBOEL [45] and UEO [47] algorithms, are considered as the optimization methods for finding the best similarity metric between the moving and fixed images. To match the previous work, we refer to the parameter settings in [42, 45], which include a population size of 30, a maximum number of generations of 100, a rotation angle in the range of [0, 360] and a translation in the range of [−30,30]. The middle slices of the CT and MR images are taken as the moving and fixed images, respectively.

In addition, we choose a similarity transformation as the experimental model, where four parameters in the matrix must be optimized, and a total of 41 different multimodal registration scenarios are taken to determine experimental results. For each registration scenario, we perform 20 independent runs of each algorithm. In particular, we select the mean NMI and RMSE values as evaluation indices. Notably, higher NMI values and lower RMSE values represent better results in the experiments. Figure 5 illustrates the final multimodal medical image registration results obtained using six different metaheuristic algorithms.

**Table 6:** Comparison of the average NMI and RMSE results of six different algorithms. The bests are highlighted in bold

Scenario	NMI					
	BSD	BBOEL	DA	GWO	UEO	NVSA
CT vs. PD (P001)	0.2379	0.2539	0.2482	0.1840	0.2537	**0.2705**
CT vs. T1 (P001)	0.2357	0.2516	0.2330	0.2317	0.2531	**0.2714**
CT vs. T2 (P001)	0.1889	0.1990	0.1875	0.1840	0.1974	**0.2027**
CT vs. PD (P002)	0.2447	0.2627	0.2463	0.2373	0.2561	**0.2662**
CT vs. T1 (P002)	0.2403	0.2298	0.2411	0.2332	0.2511	**0.2576**
CT vs. T2 (P002)	0.1985	0.2093	0.2041	0.1943	0.2083	**0.2137**
CT vs. T1 (P003)	0.2689	0.2833	0.2695	0.2399	0.2814	**0.2936**
CT vs. T2 (P003)	0.2081	0.2050	0.2128	0.2054	0.2249	**0.2332**
CT vs. PD (P004)	0.2703	0.2790	0.2681	0.2574	0.2820	**0.2863**
CT vs. T1 (P004)	0.2491	0.2626	0.2528	0.2389	0.2615	**0.2672**
CT vs. T2 (P004)	0.2065	0.2198	0.2136	0.2115	0.2224	**0.2239**
CT vs. PD (P005)	0.2273	0.2467	0.2387	0.2198	0.2386	**0.2639**
CT vs. T1 (P005)	0.2260	0.2441	0.2241	0.2188	0.2326	**0.2566**
CT vs. T2 (P005)	0.2065	0.2198	0.2001	0.1951	0.2133	**0.2239**
CT vs. PD (P006)	0.2333	0.2554	0.2349	0.2338	0.2507	**0.2606**
CT vs. T1 (P006)	0.2322	0.2513	0.2387	0.2232	0.2441	**0.2556**
CT vs. T2 (P006)	0.2000	0.1950	0.1989	0.1957	0.2075	**0.2123**
CT vs. PD (P007)	0.2602	0.2677	0.2498	0.2449	0.2684	**0.2848**
CT vs. T1 (P007)	0.2355	0.2583	0.2458	0.2390	0.2597	**0.2735**
CT vs. T2 (P007)	0.2095	0.2119	0.2136	0.2120	0.2257	**0.2333**
CT vs. PD (P101)	0.2084	0.2000	0.2033	0.2013	0.2173	**0.2222**
CT vs. T1 (P101)	0.1961	0.1862	0.1990	0.1977	0.2121	**0.2166**
CT vs. T2 (P101)	0.1755	0.1800	0.1704	0.1631	0.1796	**0.1857**
CT vs. PD (P102)	0.2127	0.1953	0.2101	0.2079	0.2177	**0.2232**
CT vs. T1 (P102)	0.2176	0.1580	0.2207	0.2169	0.2266	**0.2334**
CT vs. T2 (P102)	0.1829	0.1435	0.1739	0.1761	0.1850	**0.1902**
CT vs. PD (P103)	0.2282	0.1538	0.2203	0.2370	0.2423	**0.2427**
CT vs. T1 (P103)	0.2167	0.1499	0.2135	0.2246	0.2346	**0.2369**
CT vs. PD (P104)	0.2341	0.2544	0.2208	0.2398	0.2557	**0.2591**
CT vs. T1 (P104)	0.2367	0.2548	0.2335	0.2360	0.2567	**0.2610**
CT vs. T2 (P104)	0.2061	0.2176	0.2018	0.1981	0.2201	**0.2233**
CT vs. T1 (P105)	0.1991	0.2060	0.1968	0.1981	0.2053	**0.2082**
CT vs. T2 (P105)	0.1706	0.1749	0.1680	0.1681	0.1798	**0.1818**
CT vs. T1 (P106)	0.2132	0.1581	0.2166	0.2338	0.2360	**0.2368**
CT vs. T2 (P106)	0.1853	0.1832	0.1883	0.1940	0.2047	**0.2062**
CT vs. T1 (P107)	0.1956	0.1568	0.1974	0.1975	0.1989	**0.2001**
CT vs. T2 (P107)	0.1742	0.1772	0.1753	0.1716	0.1776	**0.1800**
CT vs. T1 (P108)	0.2092	0.1912	0.2064	0.2115	0.2168	**0.2193**
CT vs. T2 (P108)	0.1813	0.1677	0.1787	0.1833	0.1895	**0.1925**
CT vs. T1 (P109)	0.2132	0.1846	0.2103	0.1970	0.2252	**0.2303**
CT vs. T2 (P109)	0.1775	0.1740	0.1753	0.1642	0.1848	**0.1960**
Average	0.2150	0.2115	0.2147	0.2102	0.2268	**0.2341**
Friedman’s mean rank	2.63	3.05	2.63	2.02	4.66	6
Rank	4	3	4	6	2	1

Scenario	RMSE					
	BSD	BBOEL	DA	GWO	UEO	NVSA
CT vs. PD (P001)	30.983	30.928	31.046	33.930	30.342	**29.865**
CT vs. T1 (P001)	32.150	31.392	32.108	32.415	31.319	**30.647**
CT vs. T2 (P001)	**33.139**	33.554	34.376	33.930	33.706	34.221
CT vs. PD (P002)	34.544	34.736	34.135	**33.910**	34.356	35.075
CT vs. T1 (P002)	33.907	35.141	33.607	33.775	**33.516**	34.021
CT vs. T2 (P002)	**32.373**	34.105	32.761	35.361	34.140	35.927
CT vs. T1 (P003)	27.480	27.055	27.239	28.038	27.336	**26.974**
CT vs. T2 (P003)	26.286	28.020	26.452	26.429	**25.864**	26.155
CT vs. PD (P004)	26.313	26.109	26.496	27.411	25.596	**25.439**
CT vs. T1 (P004)	29.977	29.405	29.784	31.764	29.289	**28.494**
CT vs. T2 (P004)	30.996	30.879	**30.470**	31.249	30.748	31.006
CT vs. PD (P005)	35.831	35.703	35.814	35.485	35.488	**34.870**
CT vs. T1 (P005)	37.034	36.611	36.538	**36.204**	36.603	36.381
CT vs. T2 (P005)	30.996	**30.879**	31.596	32.792	33.186	31.006
CT vs. PD (P006)	27.771	26.903	27.086	27.274	26.627	**26.356**
CT vs. T1 (P006)	30.771	29.755	29.881	30.345	29.801	**29.029**
CT vs. T2 (P006)	36.231	37.747	**35.695**	35.873	36.331	37.080
CT vs. PD (P007)	29.371	29.866	29.801	**29.245**	29.421	30.115
CT vs. T1 (P007)	30.188	30.186	30.243	30.030	30.044	**29.809**
CT vs. T2 (P007)	30.308	31.189	**30.037**	30.595	31.270	33.133
CT vs. PD (P101)	**59.960**	64.670	62.386	61.076	60.300	60.137
CT vs. T1 (P101)	53.532	54.813	55.161	53.528	52.369	**52.361**
CT vs. T2 (P101)	**60.317**	63.726	64.632	65.427	62.433	61.956
CT vs. PD (P102)	49.270	53.669	51.798	**48.811**	49.055	49.677
CT vs. T1 (P102)	52.005	57.916	52.811	50.681	**50.493**	51.358
CT vs. T2 (P102)	63.452	**57.181**	67.361	66.000	64.874	64.423
CT vs. PD (P103)	36.034	57.019	37.617	**34.821**	35.250	35.211
CT vs. T1 (P103)	40.342	50.678	41.175	41.321	39.941	**38.891**
CT vs. PD (P104)	43.984	44.878	48.248	**42.938**	45.058	45.882
CT vs. T1 (P104)	49.713	48.358	50.353	**46.894**	48.776	49.548
CT vs. T2 (P104)	**53.832**	54.370	55.708	55.994	53.918	54.434
CT vs. T1 (P105)	47.850	**46.734**	48.626	47.088	47.584	47.291
CT vs. T2 (P105)	**51.845**	53.674	55.673	54.409	52.756	52.053
CT vs. T1 (P106)	53.165	61.415	53.742	**51.176**	51.369	51.181
CT vs. T2 (P106)	**60.899**	61.679	64.310	64.673	62.950	62.300
CT vs. T1 (P107)	**49.387**	54.494	49.628	50.285	49.977	50.188
CT vs. T2 (P107)	51.916	54.696	55.058	58.583	53.218	**47.755**
CT vs. T1 (P108)	47.089	50.464	48.054	46.269	**45.817**	45.895
CT vs. T2 (P108)	**53.965**	55.935	55.958	56.407	55.367	55.332
CT vs. T1 (P109)	49.716	55.211	52.836	51.730	49.663	**48.678**
CT vs. T2 (P109)	59.365	59.065	61.643	59.027	59.838	**58.017**
Average	41.812	43.678	42.877	42.517	41.853	**41.663**
Friedman’s mean rank	3.27	3.98	4.37	3.76	2.9	2.73
Rank	3	5	6	4	2	1

Table 6 demonstrates the average NMI and RMSE results obtained for “patient-001” to “patient-109” when optimized by the six algorithms.

Concerning the NMI, the NVSA rank first (as highlighted in bold) among all presented benchmarks and in terms of the average value, which is taken over 41 scenarios in total. In addition, the proposed algorithm also successively ranks first in the Friedman’s mean rank test [33]. Regarding the RMSE, the NVSA also obtains the lowest average value compared to those of the other five algorithms. The results suggest that the NVSA is the most effective algorithm since it produces the lowest ranks in the Friedman test.

**Fig. 6:**
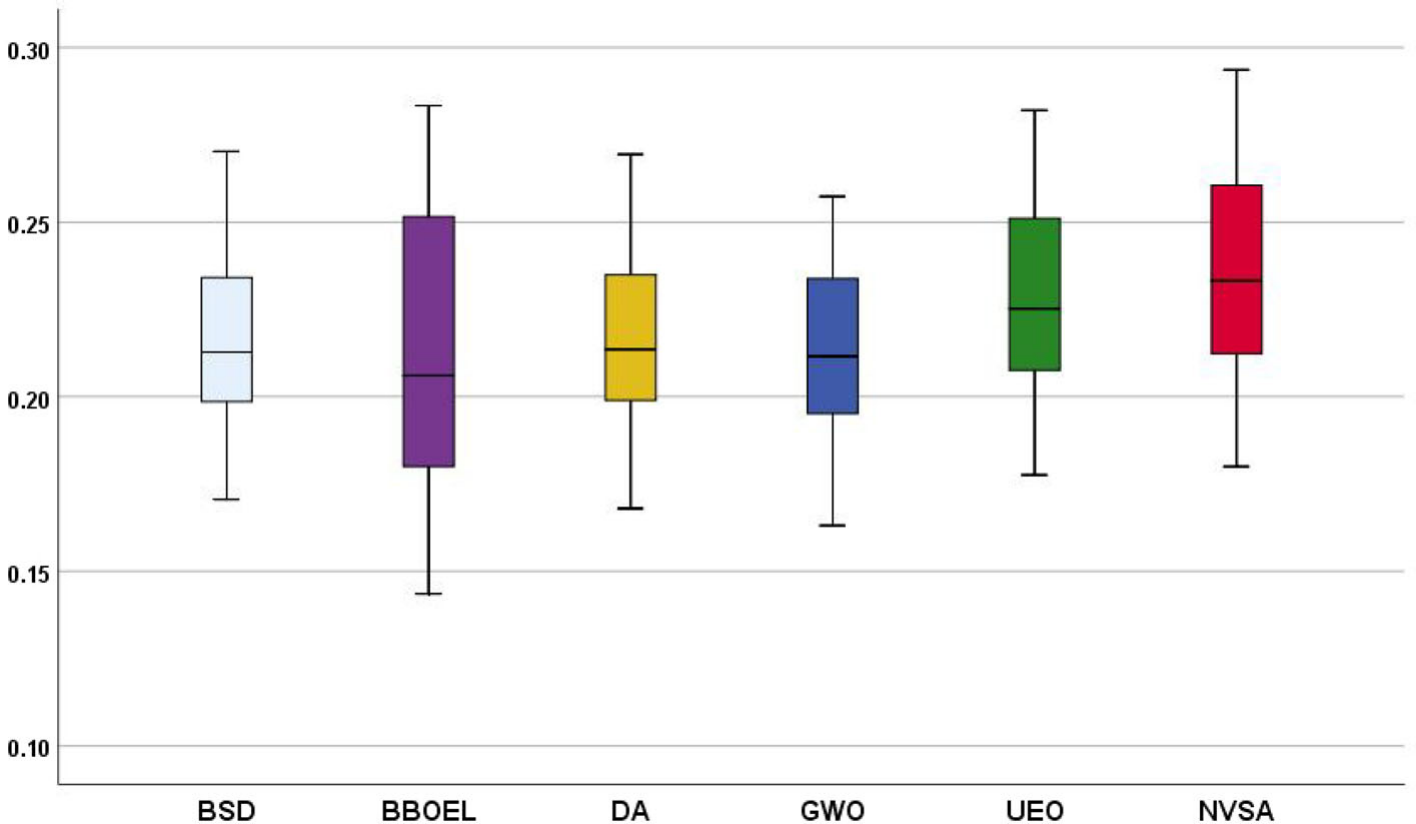
The NMI value of total 41 different multimodal registration scenarios optimized by six different algorithms

Furthermore, the boxplots statistically generated by these six algorithms are shown in Fig. 6. Five-number summaries show that the maximum, first-quartile, median, third-quartile, and minimum NMI values calculated by the NVSA are statistically higher than those of other five algorithms. These boxplots also indicate that our method significantly outperforms the other five metaheuristic algorithms.

Table 8 shows the results of pairwise comparison tests of significance, and the p-values of both the asymptotic significance (Sig) and Bonferroni correction-adjusted significance (Adj. Sig.a) metrics are less than 0.05, indicating that the results support the alternative hypothesis over the null hypothesis. The NVSA is found to be statistically significantly different from and more effective than the other five algorithms in terms of multimodal medical image registration based on the RIRE dataset. It is worth mentioning that UEO is statistically significantly different than the other algorithms except for the NVSA. This proves that UEO is also an effective algorithm, but not as effective as the NVSA.

**Table 7:** Pairwise comparison of significance test

Algorithm 1- Algorithm 2	Test statistic	Std. test statistic	Sig	Adj. Sig.a
GWO-BSD	0.61	1.476	0.14	1
GWO-DA	0.61	1.476	0.14	1
BSD-DA	0	0	1	1
BSD-BBOEL	$-$0.415	$-$1.003	0.316	1
DA-BBOEL	0.415	1.003	0.316	1
GWO-BBOEL	1.024	2.479	0.013	0.198
UEO-NVSA	$-$1.341	$-$3.247	0.001	0.018
BBOEL-UEO	$-$1.61	$-$3.896	0	0.001
BSD-UEO	$-$2.024	$-$4.899	0	0
DA-UEO	$-$2.024	$-$4.899	0	0
GWO-UEO	$-$2.634	$-$6.375	0	0
BBOEL-NVSA	$-$2.951	$-$7.142	0	0
BSD-NVSA	$-$3.366	$-$8.146	0	0
DA-NVSA	$-$3.366	$-$8.146	0	0
GWO-NVSA	$-$3.976	$-$9.622	0	0

We also demonstrate the time consumption levels of these six algorithms in six scenarios to support our analysis (Table 9). As expected, our method is competitive with the BSD, GWO, and UEO algorithms. Undisputedly, the NVSA is faster than DA and BBOEL.

**Table 8:** Quantitative time of six different algorithms

Algorithm	P001/Seconds			P101/Seconds		
	CT-PD	CT-T1	CT-T2	CT-PD	CT-T1	CT-T2
BSD	12.607	14.387	14.378	11.613	10.413	12.524
BBOEL	36.706	39.935	38.778	30.101	27.037	32.654
DA	17.906	13.012	18.293	16.473	16.664	15.972
GWO	14.134	14.080	14.137	11.163	10.492	11.512
UEO	14.137	14.113	14.094	11.672	10.540	11.415
NVSA	14.181	15.205	15.131	12.627	11.173	13.159

**Fig. 7:**
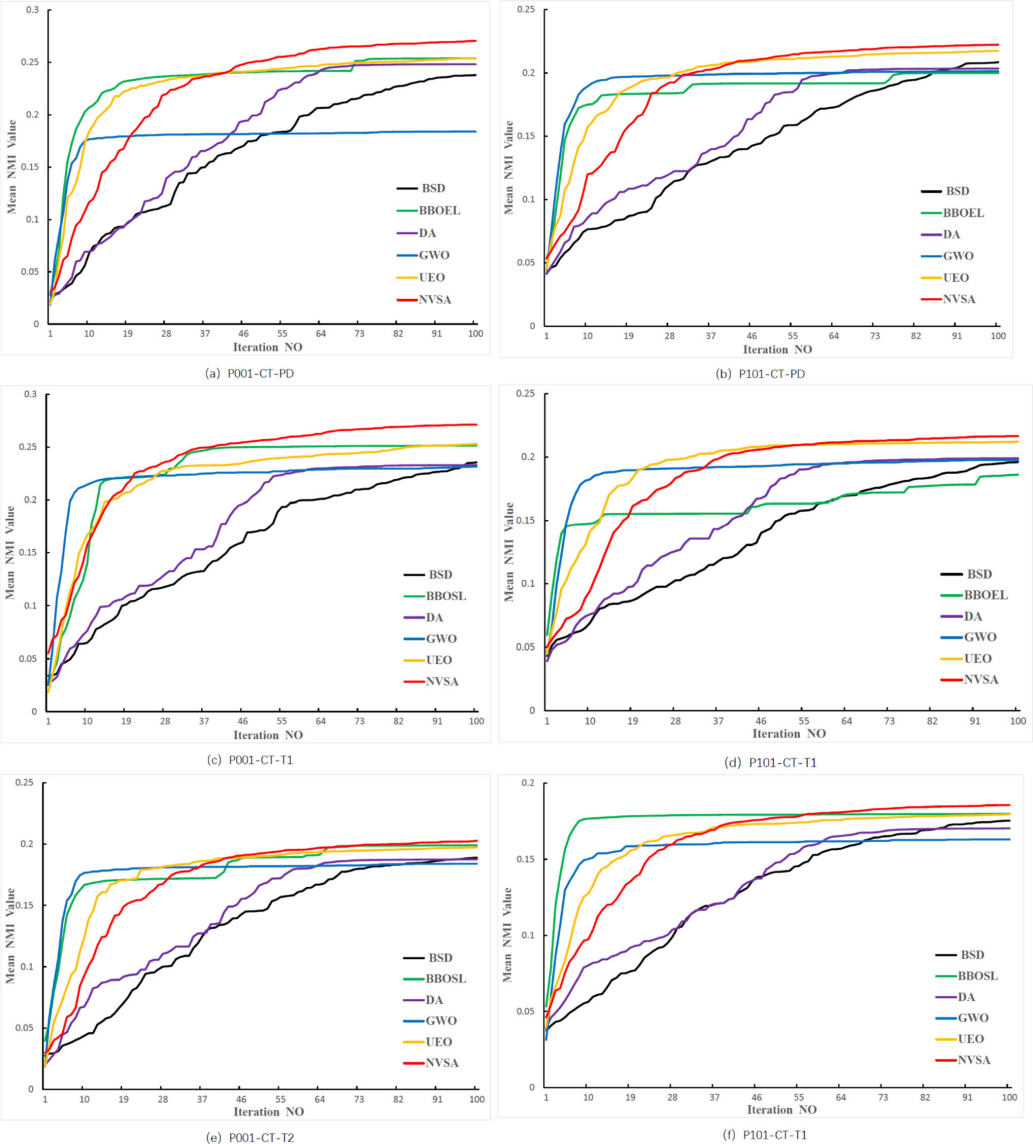
Convergence rate comparison and performance comparison with BSD, BBO-EL, DA,GWO and NVSA. Two patients with three different multimodal categories, counting in total six situations are exhibited

To more clearly present the optimization process, we also show some iterative diagrams produced during the registration process, as illustrated in Fig. 7. The two SOTA methods, UEO and BBOEL, rank second and third, respectively, in the mean NMI values obtained over a total of 41 scenarios. Additionally, including GWO, these three algorithms can converge quickly in the early stages of the iterative process compared to NVSA, but they are still slightly inferior during the later local optimization stage, whereas the NVSA can continue searching for the globally best result. This proves that the NVSA has a stronger exploitation ability than the aforementioned algorithms.

Another point worth mentioning is that the iterative NMI value of the NVSA is superior to those of DA and BSD from the beginning to the end, as shown in all six iteration graphs. It can be claimed that the NVSA attains strong exploration and exploitation abilities by modifying its search strategy and utilizing the mutation pattern derived from BSD.

### Comparisons with classic methods

We notice a lack of comparisons between classic methods and metaheuristic algorithm-based methods. In this part, three classic methods including ANTS [27, 30], FSL [29, 32], and Elastix [28, 31] are benchmarked with the proposed method with and without its initial spatial transformation (the NVSA (IST) and the NVSA, respectively) to compare their two-dimensional affine image registration performance. Notably, the metaheuristic algorithms without the initial transformation matrix easily fall into local extrema when using the affine transformation, so we take the final similarity transformation parameters in the NVSA as the initial affine transformation parameters as well as the initial spatial transformation parameters in the NVSA (IST) to estimate the rough geometric transformation. The rough registration results computed by the NVSA are also summarized.

**Fig. 8:**
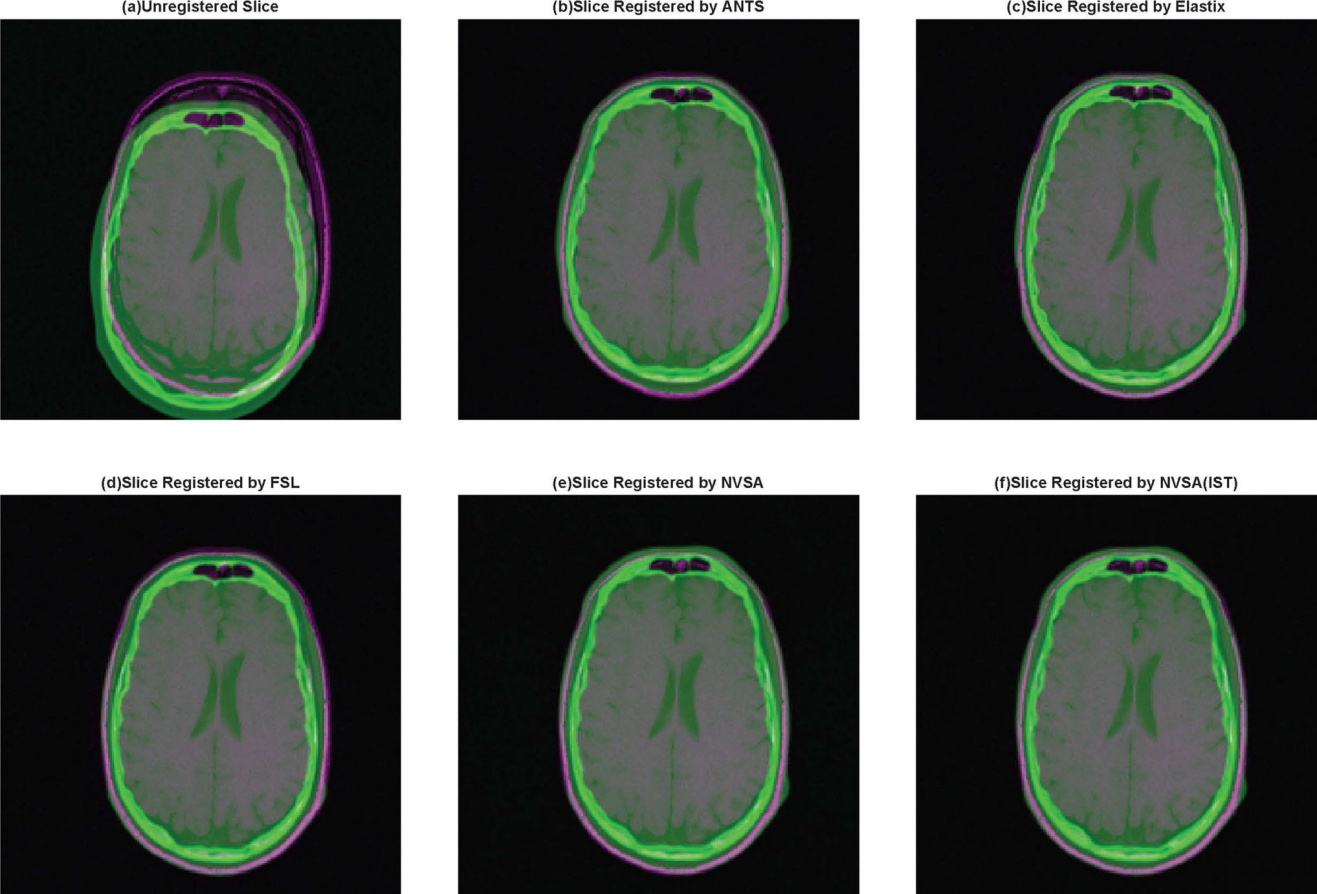
Visualizations of the registration results between multimodal P002 CT-T1 image on three classical methods and NVSA

Several changes are implemented in comparison with Section 4.2. 1) All CT images are downsampled to half their original size (256 $\times$ 256 pixels) by applying bicubic interpolation. This is because images with different sizes may not match well across these three classic methods. 2) The warped and fixed images are cut to 208 $\times$ 208 pixels because the warped images computed by these three methods contain a few blank pixels. 3) Experiments are be conducted on patients 001–007 because patients 101–109 demonstrate different blank areas than those of the former group. Taking the above factors into consideration, we remove those blank margins to make the comparison fair. Finally, we still use the NMI and RMSE values to evaluate the performance of the tested algorithm. The comparison can be seen in Table 10, and Fig. 8 illustrates the registration results obtained using different approaches from a visual perspective.

**Table 9:** Comparison of the average NMI and RMSE results of five different methods. The bests are highlighted in bold

Scenario	NMI					RMSE				
	ANTS	Elastix	FSL	NVSA	NVSA(IST)	ANTS	Elastix	FSL	NVSA	NVSA(IST)
CT vs. PD (P001)	0.3042	0.3082	0.2978	0.3081	**0**.**3114**	38.005	38.187	38.582	37.109	**36**.**127**
CT vs. T1 (P001)	0.3054	0.3036	0.2858	0.301	**0**.**311**	39.227	38.564	40.066	37.427	**37**.**084**
CT vs. T2 (P001)	0.229	0.2341	0.219	0.2324	**0**.**2388**	42.949	43.178	40.944	41.93	**40**.**902**
CT vs. PD (P002)	0.3121	0.3137	0.3194	0.3093	**0**.**324**	43.87	44.212	**42**.**474**	43.72	42.85
CT vs. T1 (P002)	0.2963	0.2907	0.2931	0.2934	**0**.**3019**	42.481	41.86	41.095	41.538	**40**.**916**
CT vs. T2 (P002)	0.2484	0.2473	0.2541	0.2491	**0**.**2583**	46.103	48.45	**43**.**242**	45.778	43.95
CT vs. T1 (P003)	0.327	0.3014	0.3305	0.333	**0**.**3491**	47.421	36.916	40.12	**33**.**224**	38.948
CT vs. T2 (P003)	0.285	**0**.**2932**	0.2882	0.2707	0.2856	33.762	33.022	32.104	**31**.**202**	31.547
CT vs. PD (P004)	0.3384	0.342	0.3305	0.3388	**0**.**3595**	31.606	31.837	32.454	**31**.**003**	32.226
CT vs. T1 (P004)	0.314	0.3148	0.3088	0.3145	**0**.**3341**	36.662	35.611	36.209	**34**.**804**	35.37
CT vs. T2 (P004)	0.2705	0.2735	0.2535	0.267	**0**.**2876**	37.57	38.191	39.793	37.888	**36**.**836**
CT vs. PD (P005)	0.3164	0.3251	0.2934	0.3092	**0**.**33**	44.495	44.187	**41**.**674**	42.744	43.342
CT vs. T1 (P005)	0.3224	0.3132	0.2846	0.2882	**0**.**3245**	45.203	45.058	45.083	**42**.**911**	45.141
CT vs. T2 (P005)	0.2599	0.2665	0.2392	0.2392	**0**.**273**	44.902	46.027	41.153	**40**.**639**	42.102
CT vs. PD (P006)	0.3064	0.3129	0.3104	0.299	**0**.**3136**	32.874	33.357	34.159	**31**.**956**	33.334
CT vs. T1 (P006)	0.2997	0.3001	0.2997	0.2923	**0**.**3047**	37.782	35.953	38.756	**35**.**222**	37.97
CT vs. T2 (P006)	0.2521	0.256	0.2552	0.2478	**0**.**2601**	45.604	45.846	44.796	44.984	**43**.**823**
CT vs. PD (P007)	0.3313	0.3381	0.3072	0.3276	**0**.**3473**	38.332	38.055	**36**.**285**	37.551	37.22
CT vs. T1 (P007)	0.3257	0.317	0.3041	0.3122	**0**.**3274**	37.96	37.668	38.594	**36**.**488**	37.635
CT vs. T2 (P007)	0.2717	0.274	0.2609	0.27	**0**.**2836**	42.798	43.761	**39**.**192**	40.301	41.142
Average	0.2958	0.2963	0.2868	0.2901	**0**.**3063**	40.48	39.997	39.339	**38**.**421**	38.923
Friedman’s mean rank	2.65	3.4	1.95	2.1	4.9	3.95	3.8	3.1	1.9	2.25
Rank	3	2	5	4	1	5	4	3	1	2

The registration results yielded by different methods are reported in Table 10. Overall, it is can be seen that compared to the commonly used methods such as ANTS, FSL, and Elastix, the NVSA (IST) rank first in terms of the Friedman’s mean rank test results and the average NMI values, and the NVSA without the initial spatial transformation ranks first in terms of the Friedman’s mean rank test results and the average RMSE values.

To be specific, the NVSA outperforms the other methods with respect to the NMI for most multimodal registration scenarios expect CT vs. T2 (P003). Regarding the RMSE, the NVSA and the NVSA (IST) rank first in 15 out of 20 scenarios. They are followed by FSL, which ranks first in 5 out of 20 scenarios. The above findings indicate that the proposed method generalizes better than the classic methods, and thus represents that the NVSA is more successful than the NVSA (IST) in comparing multimodal errors and measuring the distances between corresponding features for the RIRE dataset. Another advantage of the metaheuristic algorithm-based approach is that different transformation metrics can be obtained in each independent operation without specifically setting different initial parameters.

**Fig. 9:**
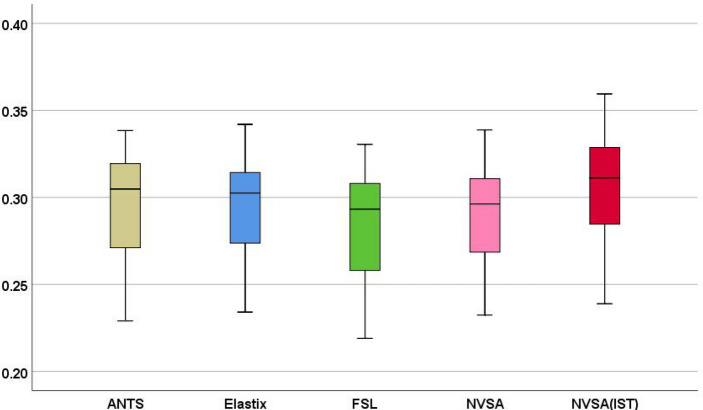
Analyzed performance of related methods for P001–P007 as mean NMI values

When examining Fig. 9, it can be said that the NVSA (IST) performs best among all five methods. In general, the proposed method is superior to the other methods according to their five-number summaries, which include the maximum, first-quartile, median, third-quartile, and minimum NMI values.

Interestingly, the NVSA and FSL do not perform well in terms of NMI (rand 4th and 5th), but they are ranked in the top three with respect to the RMSE. The reason for this may be that FSL chooses the rigid body transformation while the NVSA chooses the similarity transformation rather than the affine transformation employed by other methods. Considering the different properties of the rigid and affine transformations, specifically, the affine transformation distorts the moving image. This makes it more suitable for the NMI, which uses information entropy, than for the RMSE, which uses the distance between the corresponding features as the evaluation index.

## Conclusion

This paper presents a modified version of the BSD algorithm, called the NVSA, which incorporates an innovative normal vibration distribution, a novel mutation pattern and a new search strategy to enhance its performance. The study evaluates the proposed method on 23 classic optimization problems and 41 multimodal image registration scenarios. The results show that the NVSA outperforms the SOTA metaheuristic methods (BBOEL and UEO) and achieves better results than its predecessor (BSD) and other high-performance algorithms (such as DA and GWO). The paper presents figures, boxplots, tables and significance tests to support its conclusions, demonstrating the NVSA’s excellent exploration and exploitation abilities and its competitive convergence speed. Comparisons with three classic methods further validate the potential of the proposed algorithm for addressing real-world medical imaging needs. Future research can focus on applying the proposed metaheuristic algorithm to a 3D rigid transformation model and exploring multiobjective algorithms to find a balance between various similarity measures. The combination of metaheuristic algorithms and deep learning for coarse-to-fine registration is another interesting point worth studying.

## Data Availability

Data openly available in a public repository. The data that support the findings of classic benchmark functions are openly available in PlatEMO at https://github.com/BIMK/PlatEMO. And the multimodal human brain images are openly available in R.I.R.E. at https://rire.insight-journal.org.
